# Effects of Dietary and Lifestyle Interventions on Liver, Clinical and Metabolic Parameters in Children and Adolescents with Non-Alcoholic Fatty Liver Disease: A Systematic Review

**DOI:** 10.3390/nu12092864

**Published:** 2020-09-19

**Authors:** Christina N. Katsagoni, Eleftheria Papachristou, Amalia Sidossis, Labros Sidossis

**Affiliations:** 1 Department of Nutrition & Dietetics, School of Health Sciences and Education, Harokopio University, 70 El. Venizelou str, 176 71 Athens, Greece; Christina.katsagoni@gmail.com (C.N.K.); papachristou.eleftheria@gmail.com (E.P.); 2 Department of Kinesiology and Health, Division of Life Sciences, School of Arts and Sciences, Rutgers University, The State University of New Jersey, New Brunswick, NJ 08901, USA; 3 Athens Medical School, National and Kapodistrian University of Athens, 75 Mikras Asias str., 11527 Athens, Greece; amaliasidossis@hotmail.com

**Keywords:** NAFLD, non-alcoholic fatty liver disease, diet, lifestyle, insulin resistance, childhood obesity, children

## Abstract

Non-alcoholic fatty liver disease (NAFLD) affects 5.5–10.3% of children worldwide, while in obese individuals, it increases to almost 34%. Pediatric NAFLD is consistently associated with metabolic syndrome and insulin resistance. As no pharmacological agents exist for the treatment of NAFLD, lifestyle modifications remain the only therapy. However, as not all overweight/obese children have NAFLD, high-quality data, focused exclusively on NAFLD population are needed. Therefore, the present systematic review assessed the efficacy of lifestyle (diet or exercise) based on randomized controlled clinical trials (RCTs) on liver, anthropometric, glucose, and lipid parameters in children, with imaging or biopsy-proven NAFLD. In general, the results were inconclusive and therefore no specific recommendations could be drawn. In most studies, differences were derived from within group comparisons, which are known to be highly misleading. However, both low-carbohydrate and low-fat diets could benefit liver outcomes, as long as weight loss is achieved, but not necessary glucose and lipid parameters. No RCTs were found on exercise alone, as compared to no intervention on pediatric NAFLD. Concerning diet plus exercise interventions, all studies led to improvements in liver outcomes accompanied with weight loss. Resolution of NAFLD was found in considerably high percentages, while improvements were also seen in glucose but were modest in lipid parameters.

## 1. Introduction

Pediatric non-alcoholic fatty liver disease (NAFLD) encompasses a wide clinical spectrum of liver abnormalities associated with fat accumulation in the liver, in the absence of inherited metabolic disorders or exposure to toxic factors, including alcohol [1,2]. This diverse phenotype starts with a simple steatosis (non-alcoholic fatty liver, NAFL) and might progress to non-alcoholic steatohepatitis (NASH), with or without fibrosis. In some adolescents it might even reach end-stage liver disease [2,3]. It is estimated that NAFLD affects 5.5–10.3% of children worldwide [4,5,6]. However, this prevalence increases to almost 34% in obese children, suggesting a close relationship between NAFLD and metabolic syndrome. Insulin resistance (IR) is considered to be both a cause and a consequence of hepatic steatosis [4,6,7]. This is of particular interest, as emerging data associated pediatric NAFLD to an increased risk of morbidity and mortality in adulthood [8], as well as with an increased risk for the development of cardiovascular disease and type 2 diabetes [4].

Diet is shown to act as a risk factor in pediatric NAFLD [9]. Nevertheless, it is unclear which dietary components affect the development and progression of NAFLD in children [10]. Based on epidemiological data, obese children with NAFLD seem to have higher intakes of fructose, saturated fat, as well as the lower fiber intakes compared to those without NAFLD [9,11]. Concerning dietary patterns, in a population-based cohort study of adolescents, a close adherence in a Western dietary pattern at 14 years was associated with an increased risk of NAFLD at 17 years, an association that was dependent on BMI [12]. Moreover, leisure-time physical activities were shown to be significantly lower, while time spent in sedentary activities were higher in obese youth with NAFLD, compared to their lean or obese counterparts without NAFLD [13].

Given that no FDA-approved pharmacologic agents exist for the treatment of pediatric NAFLD [8,14], therapeutic protocols focus on lifestyle modifications for the alleviation of disease-related risk factors, namely obesity, insulin resistance, dyslipidemias, and hypertension [4,8]. Standard recommendations for all overweight and obese NAFLD children [2] aim at weight management and increased physical activity [15,16]. However, data regarding appropriate weight management protocols, optimal macronutrient content of the diet, appropriate duration of intervention, as well as exercise-related data concerning the type, intensity, and volume of exercise, remain unexplored.

Moreover, although NAFLD is closely related to obesity, not all overweight/obese children develop NAFLD [17]. One reason is that BMI used for the definition of obesity, does not take into account adipose tissue and fat distribution [18] Indeed, it is shown that ectopic fat deposition in the liver is a stronger predictor of metabolically unhealthy obese phenotype, compared to other ectopic fat deposition locations [19]. However, not all obese individuals expand their subcutaneous adipose tissue with a parallel increase in their visceral fat content, which leads to the “metabolically healthy obese” phenotype [19]. Therefore, considering all overweight/obese children to have NAFLD is inappropriate, suggesting that a precise diagnosis of NAFLD per se is needed. Although this has been highlighted by previous published systematic reviews assessing the efficacy of lifestyle modifications on pediatric NAFLD [3,6,20,21,22], the majority of the included studies were focused on overweight or obese individuals, without a NAFLD diagnosis per se [3,6,21,22]. 

As lifestyle interventions remain the cornerstone therapeutic strategy of pediatric NAFLD, there is an obvious need for strong and detailed evidenced-based nutrition, and physical activity recommendations derived from high quality data, exclusively on NAFLD patients. Therefore, the present systematic review, based only on randomized controlled clinical trials (RCTs), assessed the efficacy of dietary and lifestyle interventions on several NAFLD-related parameters in children and adolescents with imaging or biopsy-proven NAFLD. Thereafter, we summarized the efficacy of diet or exercise interventions on liver enzymes and intrahepatic triglyceride content (IHTG), as well as on anthropometric data, glucose metabolism, and lipid profile factors in children with NAFLD.

## 2. Materials and Methods 

### 2.1. Data Sources

We performed a structured search for eligible studies using specific keywords in Scopus (www.scopus.com) and the US National Library of Medicine (PubMed.gov). In order to avoid bias from outdated data, analysis was based on studies published between 1 February 2005 and 31 May 2020. Two independent researchers (E.P. and A.S.) identified all relevant publications. The Medical Subject Heading (MeSH) keywords used were—non-alcoholic fatty liver, NAFLD, NASH, fatty liver, hepatic steatosis, non-alcoholic steatohepatitis, children, adolescents, kids, child, youth, pediatric, aerobic exercise, endurance exercise, aerobic training, endurance training, diet, nutrition, weight loss, low-fat diet, low-carbohydrate diet, or combinations of the above terms. The reference list of the retrieved articles or reviews was also used to find other relevant studies. All publications were thereafter assessed using a hierarchical approach based initially on the title, the abstract, and finally on the full manuscript.

### 2.2. Inclusion and Exclusion Criteria

Eligible studies were RCTs written in English assessing the effects of diet or physical activity on children with NAFLD, aged below or equal to 18-years old. As NAFLD diagnosis might be missed in children when relying only on alanine aminotransferase (ALT) [23], the present systematic review strictly evaluated RCTs in which NAFLD diagnosis was based either on imaging or biopsy. Imaging included ultrasound (US) or proton magnetic resonance spectroscopy (^1^H-MRS) or magnetic resonance imaging (MRI) of the liver. Liver biopsy was assessed whenever possible. The exclusion of other causes for secondary liver fat accumulation was also necessary for NAFLD definition. 

The main exclusion criteria were non-human studies, non-RCTs, studies in adult population (over 18 years of age), case-reports, review studies or meta-analyses, as well as published abstracts from scientific meetings. Studies with confirmed secondary hepatic steatosis (i.e., alcohol- or drug-induced steatosis, hepatitis C, Wilson disease, lipodystrophy, coeliac disease, hypothyroidism, starvation, parenteral nutrition, abetalipoproteinemia, as well inborn errors of metabolism) were also excluded. Finally, studies with less than 10 individuals or duration of intervention of less than 1 month, or studies in which dietary interventions were considered to be the control groups and were compared with drug/vitamin supplements designated as the intervention group, were also excluded (due to the focus on the drug/vitamin effects).

### 2.3. Data Extraction

Data from the eligible RCTs were extracted by two independent investigators (C.N.K. and E.P.) in duplicates. Disagreements were resolved by a third investigator (L.S.). Extracted data were—study design and settings, study sample, inclusion and exclusion criteria, NAFLD definition, participants’ characteristics (i.e., age and sex), type of intervention, duration of the study, primary and secondary outcomes, presence of diabetes and study completion rates.

### 2.4. Outcome Measures

The assessed outcomes included changes in the serum levels of liver enzymes [ALT, aspartate aminotransferase (AST), and γ-glutamyl transpeptidase (GGT)], changes in intrahepatic triglycerides content (IHTG) as assessed by 1H-MRS or MRS, or in liver echogenicity, based on ultrasound or improvements in liver histology (when available). Resolution of NAFLD was defined either as normalization of ALT levels (i.e., reduction of ALT levels into the normal range) or normalization of hepatic steatosis (i.e., reduction of IHTG into the normal cut-off point set by each study). 

Moreover, other assessed important outcomes were changes in anthropometric measurements [i.e., weight, body mass index (BMI), BMI z-score, and waist circumference (WC), as well as percent of weight loss], various parameters of glucose metabolism [i.e., fasting glucose and fasting insulin levels, insulin resistance using the Homeostatic Model Assessment for Insulin Resistance (HOMA-IR)], as well as improvements in dyslipidemia associated factors [i.e., total cholesterol (TCHOL), triglycerides (TG), high-density lipoprotein (HDL-C), low-density lipoprotein (LDL-C), or oxidized LDL-C (ox-LDL-C)]. 

### 2.5. Study Quality

The Cochrane Risk of Bias Tool was used to evaluate the quality of the eligible studies, based on the following items—(1) random sequence allocation, (2) allocation concealment, (3) blinding of participants, (4) blinding of personnel, (5) blinding of assessors (6) completeness of outcome data, (7) selective outcome reporting, and (8) lack of other sources of bias [24]. Each fulfilled item received +1 point to a maximal score of 8; any disagreements were resolved by consensus. Studies were categorized as high quality, if the total quality score (QS) was ≥5 or low quality if QS was <5. The overall quality of the eligible studies is shown in Supplementary Table S1. Five of the included RCTs were characterized as “high quality” and five as “low quality”. The agreement between the two reviewers for the quality assessment was 0.95.

## 3. Results

**Figure 1:**
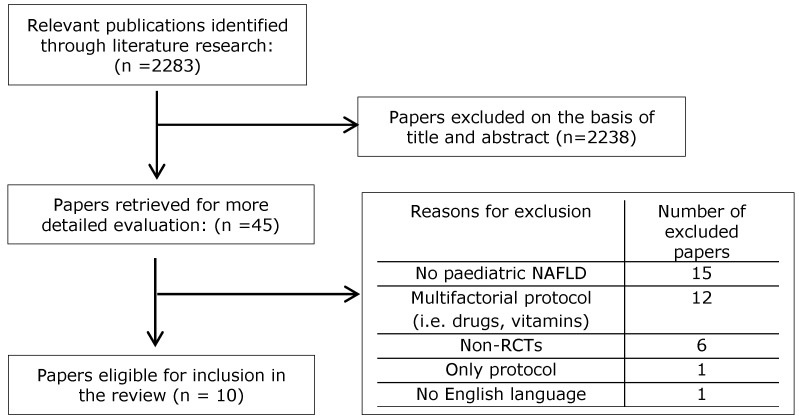
Flowchart of the included studies.

In total, 45 full-text studies were assessed for eligibility. Of those, 10 RCTs met the inclusion and exclusion criteria and were included in the present systematic review. One of the included studies [25] was a partially randomized controlled clinical trial, as the participants were randomly assigned in two of the three study groups. However, considering the protocol reasons provided by the researchers, all groups were included in the present study. The flowchart is shown in Figure 1. 

Of the eligible studies, six RCTs evaluated the effects of dietary modifications in pediatric NAFLD patients [26,27,28,29,30,31], three evaluated the effects of diet plus exercise modification [25,32,33], whereas no studies assessed exercise as the sole intervention. In this category, only one study was analyzed [34], which assessed the effects of different types of exercise, although dietary and psychological support were also given to both groups.

### 3.1. Interventions Based on Diet

Six studies [26,27,28,29,30,31] evaluated the efficacy of diet interventions on NAFLD-related parameters on 155 children with NAFLD (i.e., 79 in the intervention group and 76 in the control group), with a range of age between 9- to 18-years old. The study characteristics are shown in Table 1. The type of dietary intervention varied between studies. Two studies Ramon-Krauel et al. and Goss et al. [27,31] compared low/moderate carbohydrate diets to low/moderate fat intake diets. Two trials [26,30] compared the elimination of either fructose [26] or free sugars [30] on the diet, versus a low-fat diet or usual care, accordingly. Moreover, Jin et al. [28] provided food-based comparisons based on either fructose or glucose containing beverages. Agustini et al. [29] compared a school-meal intervention program based on a low-calorie, low-glycemic index (GI), and a low-fat diet with dietary education. 

The duration of interventions varied in these studies, from 4 weeks [28], 8 weeks [30,31], to 6-months [26,27]. Regarding the completion rates of the studies, Agustini et al. [29] and Schwimmer et al. reported 100%, Ramon-Krauel et al. [27] reported 94%, Jin et al. [28] reported 91.7%, while Goss et al. [31] reported only 78%.

#### 3.1.1. Effects of Diet on Liver Characteristics

Five RCTs [26,27,28,30,31] reported liver outcomes. The liver enzymes ALT and AST were assessed by all studies, whereas GGT was measured in only two studies [30,31]. Hepatic steatosis was defined in four studies [27,28,30,31], using ^1^H MRS [27,28], MRI [31], or MRI-proton density fat fraction (MRI-PDFF) [30]. Resolution of NAFLD or ALT was not reported in any study. Most studies reported within groups comparisons (i.e., comparisons against baseline within randomized groups). With regards to the between-groups effects, diet improved liver characteristics including ALT, AST, GGT, and IHTG, only in a study by Schwimmer et al. [30]. Within-group improvements in IHTG and ALT were also reported in the studies of Ramon-Krauel et [27] and Goss et al. [31]. 

In a study by Ramon-Krauel et al. [27], seventeen obese children were randomly assigned to either a low-glycemic diet with food-based choices of low to moderate glycemic load (GL) or a low-fat diet for 6-months. The total energy received from carbohydrates, protein, and fat in the GL group was 40%, 20–25%, and 35–40%, respectively. The low-fat diet group aimed at a total fat intake <30% of the total calories and a saturated fat intake <10%, with total energy from carbohydrates, protein, and fat being 55–60%, 20–25%, and <30%, respectively. After 6-months, both low GL and low-fat groups improved the percentage of IHTG, compared to baseline [i.e., absolute percentage change: −8.8% (standard error (SE) 4.1) versus −10.5% (3.7), respectively], although no differences between groups were achieved. The reductions in IHTG reached 37% and 36%, respectively, in each group, after 6 months. ALT levels also changed significantly in both groups, compared to baseline [i.e., low-GL group: 28.5 (9.3) IU/L; low-fat group: 18.0 (8.4) IU/L], with no significant difference between groups. No differences on the anthropometric values were found between groups.

Goss et al. [31], randomly assigned thirty-two obese children and adolescents, aged 9–17 years with NAFLD, to either a carbohydrate-restricted diet (CRD, total energy from carbohydrate:protein:fat, <25:25:>50%) or a fat-restricted diet (FRD, total energy from carbohyrate:protein:fat, 55:25:20%) in a family-based intervention program for 8 weeks. Both groups aimed at weight maintenance. At 8-week, the CRD group improved IHTG, compared to the baseline (i.e., % change in IHTG −6.0 ± 4.7%). Mean ALT levels were also reduced from 65.7 ± 54.3 U/L to 42.7 ± 27.7 U/L (mean ± standard deviation (SD)) as well as AST levels (from 48.4 ± 54.3U/L to 27.4 ± 27.7U/L) in the CRD group, compared to baseline at 8-week. No changes were observed in GGT levels in either arms of the study.

Schwimmer et al. [30], using a feeding study design, performed a study in order to test the hypothesis that free sugar restriction would reduce hepatic fat content in children with NAFLD compared to a usual diet. Forty adolescent boys aged 11 to 16 years old, were equally randomized to either the intervention diet group or the control group for 8 weeks. The dietary intervention was based on a restriction of free sugars below 3% of total energy, without restricting the total calorie intake and macronutrients. The control diet was otherwise similar to the intervention diet at baseline [i.e., moderate in carbohydrate intake, low-fat, and relatively high in protein (total energy from carbohydrate:protein:fat, 49:≥39:15%). No differences in the percentage of free sugars intake existed between groups, before intervention. At 8-week, free sugars intake was significantly reduced in the intervention group to 1%, compared to the 10% observed in the control group (adjusted week 8 mean difference (95%, confidence interval), −7.8% (−10.4% to −5.1%)). The diet low in free sugars resulted in greater reductions in hepatic steatosis in the low free-sugar diet group, compared to the usual diet group (% adjusted between group mean difference in hepatic steatosis −6.23% (95% CI, −9.45% to −3.02%), at 8-week, after adjusting for baseline values and weight change. Moreover, the mean levels of ALT, AST, and GGT were also significantly lower in the low free-sugars group, compared to the usual diet group, after adjusting for the center and baseline levels at 8-week.

Jin et al. [28] evaluated the effects of fructose reduction on overweight and obese children with NAFLD, by randomly assigning them to consume 3 servings per day of either glucose-containing beverages or fructose-containing beverages, for 4 weeks. Researchers found no changes in the % hepatic fat, ALT, and AST levels, after four weeks of reduced fructose consumption versus the isocaloric glucose diet. Similarly, six months of reduced fructose consumption compared to a low-fat diet did not affect ALT or AST levels, as found in a study by Vos et al. [26]. In both studies, there were no differences in anthropometric characteristics.

Agustini et al. [29] did not report any liver outcomes in any study groups, before or after intervention.

#### 3.1.2. Effects of Diet on Anthropometric Characteristics

Two studies [27,29] classified children as obese, in which obesity was defined as BMI ≥95th percentile for age and sex. The Centers for Disease Control and Prevention growth charts were used for this definition in both studies. As BMI varies with age and gender in children, a normalizing transformation into a z-score was considered to be necessary, by several researchers [35]. Z-scores, or standard deviation scores, describe where an observation falls within a number of standard deviations of the mean [35]. Two studies [28,31] used BMI z-score ≥85th percentile for age and sex as an inclusion criterion in their study. However, BMI z-score could be a poor metric for youth with severe obesity, considering that it did not correlate well with the adiposity levels [36]. Thereafter, both studies [28,31] examined BMI changes too, apart from just BMI z-scores, after intervention. No specific BMI cut-off points were used in a study by Schwimmer et al. [30].

Four studies [27,28,30,31] aimed at weight maintenance, but at the end of the intervention, three of those [27,30,31] reported weight loss with improvements in BMI and BMI z-scores.

Specifically, in a study by Ramon-Krauel et al. [27], in which the rationale of the study was to compare the effects of a low-GL diet versus a conventional low-fat diet, both diets were prescribed *ad libitum*. Participants only received education to eat to satiety and snack when hungry. However, at the end of the 6-month intervention, BMI decreased by (mean ± standard deviation) −1.3 ± 0.3 kg/m^2^ in the low-GL group and by −1.2 ± 0.3 kg/m^2^ in the low-fat group, compared to baseline, but these changes did not differ significantly between groups. Accordingly, BMI z-scores and WC values were also significantly decreased after intervention in both groups, compared to baseline.

As already mentioned, in a study by Goss et al. [31], researchers aimed to compare the effects of an individualized weight-maintaining CRD versus an FRD, on IHTG and insulin resistance in children and adolescents with NAFLD, after a family-based intervention for 8 weeks. Although researchers aimed at weight maintenance, after-intervention weight loss tended to be higher in the CRD group, compared to the FRD group (i.e., −2.4% vs. −0.4%, *p* = 0.06). The CRD group also experienced significant improvements in BMI compared to the FRD group (i.e., 3% vs. 0.3%, *p* < 0.05, accordingly), as well as in BMI z-scores.

Similarly, in a study by Schwimmer et al. [30], children were given weekly meal plans based on their habitual diet (according to 24-h food recalls and a 7-day food diaries). The only requirement was to keep free-sugar intake to less than 3% of their daily caloric intake, but without restricting total calorie intake and macronutrients. However, at the end of intervention, the mean weight decreased from 88.1 kg to 86.7 kg in the restricted free-sugar arm of the study, compared to an increase in weight, which varied from 88.7 kg to 89.3 kg (between-group adjusted for baseline mean difference, −2.00 kg [95%CI, −3.30 to −0.79 kg]) in the usual diet group. There were also significant differences in the BMI and z-scores between groups, at the end of intervention.

Vos et al. [26] and Jin et al. [28] reported no difference in body weight between groups, after intervention.

The duration of the intervention did not seem to affect changes in the anthropometric values. In the studies of Ramon-Krauel et al. [27] and Vos et al. [26] in which the effects of a low/moderate carbohydrate diet (i.e., either based on low-GL or low fructose intake) versus a low-fat diet were assessed after 6 months of intervention, contradictory results regarding changes in weight and BMI were found.

#### 3.1.3. Effects of Diet on Glucose Metabolism

Five out of six RCTs assessed fasting glucose parameters [27,28,29,30,31]. All measurements were at the fasting stage. In a study by Goss et al. [31], insulin was measured in serum, whereas in the studies of Jin et al. [28] and Ramon-Krauel et al. [27], it was measured in plasma. No information was given by the other studies. HOMA–IR was improved in two studies [27,31], in which the fasting insulin levels also changed significantly, while improvements in insulin sensitivity were found in one study [28]. 

The type of dietary intervention that led to glucose metabolism changes differed between studies. In a study by Ramon-Krauel et al. [27], only the low-fat group was shown to decrease fasting plasma insulin and HOMA–IR levels, compared to baseline at 6 months. No such improvements were observed in the low GL diet compared to baseline, although similar weight loss was achieved in both groups at 6 months. 

In contrast, in a study by Goss et al. [31], CRD was shown to be more effective in improving fasting serum insulin and HOMA–IR levels, compared to FRD at 8-week, while at the same time weight loss tended to be higher among CRD compared to the FRD group. 

Moreover, in a study by Jin et al. [28], the role of insulin in adipose tissue was investigated. Insulin suppresses lipolysis and promotes glucose uptake in adipose tissue, and thereafter regulates the secretion of free fatty acids into the bloodstream [37]. With this rationale, researchers calculated the adipose insulin resistance index (i.e., adipose-IR) by multiplying fasting plasma insulin levels with fasting plasma free fatty acids levels. After four weeks of reduced fructose consumption versus the isocaloric glucose diet, children had significant improvements in adipose-IR index as compared to the fructose arm of the study (mean ± SE: −28.1% ± 7.6% vs. 47.0% ± 27.6%, respectively), suggesting improved adipose-insulin-sensitivity.

Agustini et al. [29] aimed to study whether a low calorie, low GI, and low-fat diet could improve IR in children with NAFLD, compared to usual care. Of 32 subjects that enrolled in the study, 16 were randomly assigned to the intervention group and the other to the control group. The intervention group received a lunch box at school, which consisted of a low-GI and a low-fat diet (fat <25% of total calories, cholesterol <300 mg/day), as well as nutrition education for 12-weeks. No information was given about the energy deficit of the dietary intervention. The control group received only nutrition education for the same period. At the end of the study, although only the intervention group showed reductions in energy intake compared to baseline, both groups reduced carbohydrate and fat intake within groups. Researchers did not present any comparisons between groups regarding diet at 12-weeks. However, fasting insulin levels and HOMA-IR differed significantly between groups at the end of the study, but surprisingly, both insulin and HOMA-IR increased in the intervention group compared to the control group. Researchers attributed these unexpected results to the so-called “yoyo phenomenon” indicated by the weight fluctuations observed in both groups during the study. This phenomenon further resulted in no changes in weight and BMI between groups after intervention, suggesting a low adherence in the study protocol.

No significant differences in glucose, insulin, and HOMA-IR were observed between groups in a study by Schwimmer et al. [30], after intervention. Vos et al. [26] did not evaluate glucose parameters at any time and in any study group.

The duration of the study seemed to be irrelevant to glucose parameters changes, as improvements were observed after both 8 weeks [31] and 6 months [27] of intervention.

#### 3.1.4. Effects of Diet on Lipid Profile 

Five RCTs [26,27,28,30,31] evaluated the effects of dietary modification on lipid profile. In a study by Goss et al. [31], lipid profile was measured in serum, whereas in a study by Jin et al. [28], it was measured in plasma. No information was given by the other studies.

In the trials of Goss et al. [31] and Ramon-Krauel et al. [27], the lipid parameters remained unchanged in both the intervention and the control groups, at the end of the study. As previously mentioned, the improvements in the anthropometrics achieved in the intervention group, as compared to the control group in a study by Goss et al. [31] had no effect on the lipid profile of participants.

In a study by Schwimmer et al. [30], only the TCHOL levels were improved at the restricted free-sugars intervention group compared to the usual diet [i.e., between-groups adjusted mean difference −15.16 mg/dL (95%CI, −25.67,−4.65 mg/dL] at 8-week. 

Ox-LDL-C levels decreased in a study by Vos et al. [26] after 6-months of reduced fructose consumption compared to baseline, whereas no changes were observed in the low-fat group. Similarly, in a study by Jin et al. [28], 4 weeks of reduced fructose intake led to reductions in plasma ox-LDL-C levels in the low fructose group compared to baseline, but not to the low-glucose group. However, changes in ox-LDL-C in both Vos et al. [26] and Jin et al. [28] studies, were not significant between study groups. None of the other lipid parameters were reported in those three studies [26,28,30] changed after intervention.

A study by Agustini et al. [29] did not report any outcomes regarding lipid profile in any study groups, before or after intervention.

### 3.2. Interventions Based on Diet Plus Physical Activity

Three studies [25,32,33] evaluated the effects of diet plus physical activity interventions on NAFLD. Study characteristics are shown in Table 2. Dietary interventions were based either on a specific dietary plan by Wang et al. [32] or on nutrition modification sessions of Koot et al. [25] and Chan et al. [33]. Physical activity interventions were based on aerobic exercise sessions, in the studies of Wang et al. [32] and Chan et al. [33], or on high-intensity aerobic exercise, in the study by Koot et al. [25]. In total, 164 children with NAFLD (i.e., 82 in the intervention group and 82 in the control group), aged between 8 to 18-years old participated in these studies.

Duration of intervention varied between studies, from 1 month [32] to 16 weeks [33] and 6 months [25]. Finally, follow-up measurements were made in two studies [25,33], after 18 months (24 months from baseline) [25] and after 52 weeks (68 weeks from baseline) [33]. Regarding the completion rates of the studies, Koot et al. [25] reported 91% at 6 months and 80% at 24 months, while only 80.8% of the participants finished the study by Chan et al. [33].

#### 3.2.1. Effects of Diet Plus Exercise on Liver Characteristics

All studies [25,32,33] reported liver outcomes. ALT was measured in all studies [25,32,33], AST in two studies [32,33], while none of the studies assessed GGT levels after treatment. Hepatic steatosis was defined using either US [32] or ^1^H MRS [25,33]. Resolution of NAFLD was assessed by all studies and was defined as normalization of ALT levels in one study [32], or both ALT and IHTG, in the other two studies [25,33]. Two trials reported liver outcomes after 68-weeks [33] and 2 years of follow-up [25]. 

The results were mainly focused on within-group comparisons. Only Chan et al. [33] reported between-group differences in IHTG, after intervention, accompanied by Koot et al. [25], which also showed IHTG reductions, but within-groups. Both studies found these improvements to remain within-groups, after their follow-up periods [25,33]. ALT differences were also observed within groups in the studies of Wang et al. [32] and Koot el al. [25]. Of note, although resolution of NAFLD was reported by all studies [25,32,33], only Koot et al. [25] demonstrated within-group improvements at the end of intervention and at 2-years of follow-up.

Specifically, in a study by Chan et al. [33], obese NAFLD Chinese adolescents were randomly assigned either to a dietitian-led (DL) or a pediatrician-led (PL) intervention for 16 weeks and were followed up for 68 weeks. At 16-weeks, the DL group had significantly lower total and saturated fat, as well as higher protein intake and a significant increase in physical activity level, compared to the PL group. The percentage of intrahepatic triglycerides content (IHTG) had significantly reduced in the DL group compared to the PL group, after intervention (between groups mean difference (SE) −3.1% (1.36)). Regarding resolution of NAFLD (defined as presence of hepatic steatosis lower than 5% of IHTG measured by 1H-MRS), 6/22 (i.e., 27%) of adolescents in the DL group versus 4/24 (i.e., 16.6%) in the PL group, showed complete resolution of NAFLD in the 16-week evaluation. Researchers did not report whether this comparison was significant between groups, but at the same time, ALT and AST did not change significantly between groups. After 68-weeks of follow-up, the groups no longer had differences in diet and physical activity. Of note, the anthropometric improvements achieved in the DL group at the 16-week evaluation did not sustain after the follow-up period. However, IHTG improvements remained significant in the DL group compared to baseline, but not amongst the groups at follow-up. The percentage of patients with NAFLD resolution became equal between groups, without researchers reporting statistical significance. No differences were still observed in the ALT and AST levels amongst groups, at 68-weeks.

In a study by Koot et al. [25], severely obese, non-diabetic children with liver steatosis were allocated to inpatient treatment (for 2 or 6 months), ambulatory treatment (for 6 months), or usual care (for 6 months). The intervention groups received standardized exercise sessions consisting of high-intensity aerobic exercise (indoor, outdoor, and swimming activities) in groups, nutrition, and behavior modification therapies. NAFLD resolution was based on normalization of ALT levels and IHTG. With regards to IHTG, resolution was defined as IHTG < 1.8% of absolute mass concentration of liver, measured by ^1^H-MRS. Based on the study results, IHTG was significantly normalized in 43%, 29%, and 22% of patients in the inpatient, ambulatory, and usual care group at 6 months, compared to baseline, respectively. Accordingly, ALT significantly normalized in 41%, 33%, and 6%, respectively. However, researchers did not report whether these changes in both ALT and IHTG proportions were significant between groups, at 6 months. At 2 years of follow-up, IHTG and ALT normalization were present in around 40% of the inpatient group and 30% of those in the ambulatory setting, compared to baseline. No information was given for between-group comparisons at 2-years, however, these percentages were significant within both groups, regarding the IHTG normalization, but only in the inpatient group concerning ALT normalization. The control group was not assessed in follow-up.

Wang et al. [32] randomly assigned obese children with NASH to a lifestyle intervention group on a summer camp or to a control group. The intervention group received a low-calorie diet aiming at weight loss, while the subjects had to participate in 3 h of aerobic exercise activities (e.g., swimming, basketball, table tennis) every day for one month. The control group did not receive any intervention. After one month, ALT and AST levels and anthropometric parameters significantly improved in the intervention group, compared to baseline, while the control group did not achieve any improvements. Although no significant changes were observed in the liver ultrasonography measurements compared to baseline, 10/19 (i.e., 52.63%) of the individuals in the lifestyle intervention group had normal liver functions after the summer camp period was completed.

#### 3.2.2. Effects of Diet Plus Exercise on Anthropometric Characteristics

All studies [25,32,33] enrolled obese children. Obesity was defined using BMI ≥ 95th percentile for age and sex, in all studies. However, all studies reported results for the BMI z-score as well. Chan et al. [33] and Wang et al. [32] used BMI reference curves for Chinese children. Weight loss was a study goal in two trials [32,33], although the exact percent of achieved weight loss was not reported. Within-group improvements were observed in all studies. However, the between-group effects of diet and exercise on anthropometric indices were reported in only one study [33].

Specifically, in a study by Chan et al. [33], the DL group received menu plans based on USDA recommendations targeting a desired weight status, while individualized exercise programs were also given to each adolescent. On the contrary, individuals at the PL group did not receive any specific dietary or exercise plans but they were encouraged to reduce high-GL choices and animal fat intake, as well as to exercise for at least 30 min, 2–3 times a week. At the end of the 16-week intervention, weight (−4.5 kg), BMI (−1.45 kg/m2), BMI z-score (−0.16), as well as WC (3.71 cm) were improved in the DL vs. the PL groups. However, none of these improvements remained significant between groups at 68-weeks of follow-up. 

Koot et al. [25] observed minor improvements in BMI z-score in both inpatient (−0.37 kg/m^2^) and ambulatory (−0.16 kg/m^2^) groups, 6 months after intervention. After 24-months of follow-up, these changes remained only in the inpatient group (BMI z-score mean change—0.35 kg/m^2^). 

Weight loss was aimed in a study by Wang et al. [32], using an energy deficit of 250 kcal/day with a dietary plan of 1300–1600 kcal. Although the percent of weight loss was not reported, participants reduced their BMI and BMI z-score within the intervention group. No reductions in BMI and BMI z-score were observed in the control group.

#### 3.2.3. Effects of Diet Plus Exercise on Glucose Metabolism 

All studies [25,32,33] assessed glucose metabolism. Only Chan et al. [33] reported between group effects, but all studies provided within-group comparisons.

Specifically, in a study by Chan et al. [33], the DL group improved HOMA–IR (−2.00) and fasting plasma glucose levels (−0.23 mmol/L) compared to the PL group at 16-week. Insulin levels (−4.98 mIU/l) were also improved in the DL group, compared to baseline at 16-week, but without statistical significance between groups. HOMA–IR and fasting plasma glucose levels differences were no longer observed at 68-week [33]. 

Koot et al. [25] observed minor improvements in HOMA–IR, 6 months after intervention, only in the inpatient group (−1.1) compared to the baseline. These differences remained significant (−0.8) after 24 months of follow-up. 

Finally, Wang et al. [32] showed both fasting serum insulin levels (mean ± SD, 15.54 ± 4.5mIU/L to 8.53 ± 4.08 mIU/L, *p* < 0.001) and HOMA–IR (mean ± SD, 2.87 ± 0.88 to 1.63 ± 0.92, *p* = 0.002) to be significantly improved in the summer camp lifestyle group, compared to the baseline, one month after intervention. No such improvements were observed in the control arm of the trial.

#### 3.2.4. Effects of Diet Plus Exercise on Lipid Profile

All studies [25,32,33] assessed the lipid profile of participants before and after intervention. 

In a study by Koot et al. [25], serum lipid levels remained unchanged in all arms and all time points of the trial.

Similarly, Chan et al. [33], did not observe any improvements in serum lipid levels between groups at both 16 and 68-week. However, modest reductions in fasting serum TG levels were found in both groups, compared to baseline, at 16 and 68-week. Fasting serum TCHOL levels were significantly changed in the PL group (TCHOL mean ± SD difference within PL group: +0.23 ± 0.39 mmol/L), compared to baseline, at 16-week. Improvements were also seen in the fasting serum HDL-C in both groups (HDL-C mean ± SD difference within the PL-group: +0.11 ± 0.13 mmol/L vs. within DL-group: +0.10 ± 0.18 mmol/L), compared to baseline but only at 68-week.

Wang et al. [32] showed levels of fasting serum TCHOL (4.83 ± 0.92 mmol/L to 4.54 ± 0.98 mmol/L, *p* < 0.012) and fasting serum TG levels (2.87 ± 0.88 mmol/L to 1.63 ± 0.92 mmol/L, *p* = 0.002) to be significantly improved in the summer camp lifestyle group, compared to baseline, one month after intervention.

### 3.3. Interventions Evaluating the Effects of Physical Activity

We did not find any randomized controlled clinical trials on exercise as monotherapy, versus usual care in children with NAFLD. Only one study [34] evaluated the effects of different types of physical activity on NAFLD, while dietary and psychological support were also given to both groups.

Indeed, de Piano et al. [34] compared the effects of aerobic training (AT) with those of aerobic plus resistance training (AT+RT) in NAFLD obese adolescents. Participants in the AT group followed an 180 min per week (i.e., 60 min × 3 times) routine of aerobic exercise, whereas individuals in the AT+RT group performed 30 min of aerobic exercise and 30 min of resistance exercise, three times per week, for one year. Both groups received the same nutritional (i.e., weekly dietetic lessons providing valuable information on healthy diet, e.g., the food pyramid, weight-loss diets, etc.) and psychological intervention (i.e., weekly psychological support group sessions, e.g., discussions about body image, binge eating, etc.); although differences between studies regarding diet and psychology were not evaluated after intervention. At the end of the study, both groups had lower BMI and body mass levels compared to baseline, although it was not mentioned if there were differences between groups. However, researchers observed that the AT+RT group also showed lower levels of serum ALT (but not of AST), fasting serum insulin, and HOMA–IR, fasting serum TCHOL and LDL-C after one year of intervention, compared to AT.

## 4. Discussion

The present systematic review only evaluated the RCTs related to lifestyle interventions, i.e., diet or exercise in children with imaging or biopsy-proven NAFLD. As not all overweight/obese children have NAFLD [17], the current review focused exclusively on the NAFLD population and not just overweight or obese individuals without an NAFLD diagnosis per se. This further highlights the novelty of current review, as most of the existing reviews were not conducted exclusively on NAFLD patients [3,6,21,22]. 

Overall, 10 RCTs evaluating the effect of dietary and lifestyle interventions on liver and other clinical and metabolic factors in children with NAFLD, were found in the literature. Considerable heterogeneity was found amongst studies regarding the duration and type of intervention, as well as their outcomes. The studies that focused on dietary modification did not identify an optimal dietary treatment for the management of children and adolescents with NAFLD. No RCTs were found that examined the effects of exercise alone, compared to no intervention on pediatric NAFLD patients. The only RCT of de Piano et al. [34] that compared two different types of exercise found that aerobic plus resistance exercise was superior to aerobic exercise alone. RCTs of diet plus exercise were also limited. Nevertheless, most of the existing studies either evaluated the effects of diet, diet plus exercise, or exercise alone. Differences were mainly derived from within-group comparisons, which are known to be highly misleading [38]. Indeed, comparisons against baseline are considered to be invalid and might lead to an inflated type I error and misinterpretation of the study results [39]. This is because type I error depends on the power of the paired tests to detect a true change from baseline within each group rather than the effect of the intervention on the outcome’s change over time [38,39]. Therefore, there is a greater need for more high-quality studies assessing the effects of lifestyle in pediatric NAFLD. 

Concerning the studies focused on dietary modification, they used different dietary protocols, and therefore, conclusions regarding the optimal macronutrient content of the diet could not be made. As an example, in experimental studies, fructose appeared to have a major role in inducing fatty liver [40]. However, reducing fructose intake does not always improve liver parameters, as it was also shown in the studies of Jin al [28] and Vos et al. [26]. However, when total free sugars (i.e., glucose, fructose, and sucrose) were reduced below 3%, as in a study by Schwimmer et al. [30], liver and other metabolic improvements were observed. Even modest reductions in fructose intake (i.e., <7% of total energy intake) or in GI (i.e., 44–55) and GL (i.e., <80) resulted in improvements of liver function and cardiometabolic risk factors in childhood NAFLD [41]. Indeed, according to the World Health Organization [42], free sugar intake should be limited to less than 10% per day for all people, and should be even lower in specific circumstances. Nevertheless, improvements were also observed when a low-fat diet was adopted by children with NAFLD, as in a study by Ramon-Kraouel et al. [27]. Based on data in adults, both low/moderate carbohydrate and low/moderate fat intake seemed to provide similar results on liver function [43], suggesting that other factors such as the duration of the intervention or the magnitude of weight loss might play a pivotal role in the management of NAFLD.

Indeed, although weight loss is considered to be a standard recommendation for all overweight and obese NAFLD children, only one study among those on dietary modification, aimed at weight loss [29]. However, minimal improvements were observed in weight, BMI, and BMI z-scores, either between- or within-groups, in studies by Schwimmer et al. [30], Ramon-Krauel et al. [27], and Goss et al. [31], after intervention. Weight loss accompanied with improvements in liver outcomes (i.e., IHTG and ALT levels) in all studies, but not in glucose metabolism (only improved in Ramon-Krauel et al. and Goss et al.) and lipid profile (only improved in Schwimmer et al.). Therefore, as already shown in adults [43], both low-carbohydrate and low-fat diets could benefit children with NAFLD considering liver outcomes (namely IHTG and ALT), as long as weight loss was achieved, but this did not necessary lead to glucose and lipid improvements.

The exact degree of BMI reduction or weight loss required to improve liver as well as other NAFLD-related parameters in those children is not known. Similarly, whether weight maintenance could benefit children with NAFLD still has to be elucidated [3]. For instance, Jin et al. [28] and Vos et al. [26] did not report any significant improvements in anthropometrics parameters within groups at the end of their studies. However, both showed reductions in ox-LDL-C (although not in liver parameters), which is known to be strongly associated with induction of oxidative stress, venous inflammation and fibrosis, leading to the progression of NAFLD in both adults [44] and children [45]. Whether diet could thereafter delay the progression of NAFLD, even without weight loss, in children with NAFLD is unclear, but it could be really helpful for those NAFLD children who could not lose weight. Nevertheless, in adults with NAFLD, a weight loss of ≥10% is needed to accomplish a complete NASH resolution, whereas even modest weight reductions were proven to be beneficial in improving hepatic steatosis and histological indices [46,47]. However, since children grow at a slow rate, long-term studies (1–2 years) are needed to assess the effects of various interventions of body weight management.

Furthermore, whether specific dietary patterns are required to achieve these improvements in weight status to benefit NAFLD is not known in children with NAFLD. Data derived from adults clearly highlight the beneficial effects of the Mediterranean diet on liver metabolism [43,48]. No such interventional data exist in children. Based on few epidemiological data conducted in a pediatric population with NAFLD, low adherence to the Mediterranean diet was associated with higher likelihood of having NASH, as well as higher values of C-reactive protein, fasting insulin, and HOMA–IR, compared to high adherence [49].

Moreover, although exercise is universally recommended for the treatment of NAFLD, endorsed by NASPGHAN [15] and AASLD [16], no randomized clinical trials on exercise alone versus usual care were found in children with NAFLD. Therefore, current recommendations for pediatric NAFLD patients are the same as for all children. The World Health Organization [50] recommends 60 min of moderate to vigorous-intensity physical activity daily, while incorporating at least 3 days per week of muscle-strengthening physical activities. Still, the effects of the duration of exercise, type, intensity, and volume on pediatric NAFLD need to be elucidated, as well as whether these effects occur even in the absence of weight loss.

Nevertheless, several clinical trials that were conducted in obese pediatric populations (without diagnosis of NAFLD per se) demonstrate positive results of physical activity on liver characteristics and metabolic factors [51,52,53,54]. In a meta-analysis [55] of 14 RCTs in overweight and obese children, Gonzalez-Ruiz et al. [55] examined the effectiveness of exercise interventions on liver enzymes and IHTG. Exercise versus usual care was associated with significant reductions on GGT and IHTG levels, while no further sensitivity analysis was performed regarding the effect of the type of exercise (i.e., aerobic versus resistance exercise or combination). The study was clearly based on obese pediatric patients without a diagnosis of NAFLD per se, therefore, no specific recommendations for NAFLD patients could be drawn. Nevertheless, as discussed above, the RCT of de Piano et al. [34] showed that 180 min of aerobic plus resistance training was superior to 180 min aerobic training on ALT and several other clinical and metabolic factors. However, dietary and physiological support were also given to both groups. 

Meta-analyses in adult population with NAFLD clearly showed that exercise alone has a large effect on IHTG levels, which remains significant even in the absence of weight loss [56,57]. Both aerobic [58] and resistance exercise [59], compared to usual care or their combination [43], are shown to improve IHTG or other glucose metabolic indices, such as HOMA–IR and HbA1C, while no clear superiority of aerobic exercise over resistance exercise exists [60,61].

Furthermore, very few RCTs were found on lifestyle interventions, including dietary and exercise modifications in children with NAFLD. All studies reported weight or BMI reductions. Only Chan et al. [33] that assessed between-groups differences, showed greater improvements in IHTG and several other NAFLD-related parameters after intervention, including HOMA–IR and fasting plasma glucose levels (but not liver enzymes and lipid profile). However, none of these improvements remained significant between groups during follow-up, highlighting the need to evaluate the long-term effects of each intervention [3]. Whether this had to do with the adherence to such study protocols in general is not known. However, only 80.8% of participants in a study by Chan et al. [33] completed the trial, and even less (i.e., 80%) completed in a study by Koot et al. [25], at 2 years of follow-up. Indeed, data from obesity-related interventions in children show that the majority of obese individuals do not adhere to dietary programs, while of those who do, the majority returns to their previous weight or do not lose weight at all [62]. However, as obese children with NAFLD might progress to more serious liver conditions if left untreated, more studies are needed in order to determine the appropriate duration and the factors associated with low adherence to such therapeutic protocols.

Moreover, NAFLD resolution, namely the normalization of either IHTG or ALT levels was considerably high at end of lifestyle interventions in the studies by Wang et al. [32] and Koot et al. [25]. Only Koot et al. [25] reported significant improvements in NAFLD resolution within the inpatient and ambulatory groups at the end of study, but in lower percentages, at 2 years of follow-up. Whether the combination of diet with exercise could be superior to that of diet alone or exercise alone in pediatric NAFLD is unclear. Nevertheless, lifestyle interventions seemed to have a more pronounced effect on liver enzymes, compared to exercise alone, as shown in adults [43]. Additionally, the effects of other aspects of lifestyle, namely sleep duration and quality, which is believed to have a considerable effect on NAFLD-risk also remain unexplored in children [63,64,65].

Finally, lean patients with NAFLD seem to share the same metabolic features of insulin resistance and dyslipidemia in obese patients with NAFLD [66]. Apparently, the role of body composition on lean subjects with NAFLD is significant [67]. Lifestyle changes aiming at improving overall fitness are also likely to have a favorable impact on those patients [66]. In the present review, none of the RCTs examined had incorporated lean children with NAFLD, although this was not an exclusion criterion. On the contrary, everyone in the included studies were overweight or obese. Therefore, the efficacy of specific dietary patterns that could play a protective role against the disease, independently of BMI, needs to be explored within future studies.

## 5. Conclusions

In the present systematic review, only 10 RCTs were identified as having assessed the effects of lifestyle, i.e., diet or exercise intervention, on children with NAFLD. The current review, evaluated RCTs in which hepatic steatosis was defined on the basis of imaging or biopsy, while all studies with non-clearly defined NAFLD population were excluded. In general, the results are inconclusive and, therefore, we could not make specific recommendations regarding diet and exercise in children with NAFLD. A major limitation of the included studies is that differences were mainly focused on within-group comparisons, which are known to be highly misleading. Nevertheless, concerning the effectiveness of dietary modification for the management of pediatric NAFLD, both low-carbohydrate and low-fat diets might lead to improvements in liver outcomes (namely IHTG and ALT), as long as weight loss is achieved, but they did not necessary improve the glucose and lipid parameters. No RCTs were found that examined the effects of exercise alone, compared to no intervention on pediatric NAFLD patients. Although, only 3 RCTs were found on the efficacy of diet plus exercise in children with NAFLD, all studies led to improvements in liver outcomes accompanied with weight loss. Resolution of NAFLD was found in considerably high percentages, while improvements were also seen in glucose metabolism parameters, but were modest in lipid profile. 

Moreover, longer duration RCTs are needed to evaluate the effects of several parameters associated with lifestyle on pediatric NAFLD, namely optimal weight loss and macronutrient content of the diet, appropriate duration of intervention, the factors associated with low adherence, as well as sleep duration and quality. Regarding exercise interventions, the effects of the duration, type, intensity, and volume of exercise still remain unexplored in children with NAFLD. Additionally, more research is needed to explore the effects of lifestyle intervention in lean pediatric NAFLD patients.

## Figures and Tables

**Table 1:** Characteristic of interventions based on diet.

Author [ref]	Journal	Country	Sample (n)	Intervention Group	Control Group	Nafld Definition	Age (y) (mean ± SD)	Sex (boys)	Type of Intervention	Dura-tion	Primary Outcomes	Secontary Outcomes	Completion Rates
**Diet only interventions**													
Vos et al, 2009 [26]	Arch Pediatr Adolesc Med	USA	10	6 (Low-fructose group)	4 (Low-fat group)	Liver biopsy or serology and US	Low-fructose group: 13.3 ± 0.65. Low-fat group: 12.5 ± 1.0.	NR	Low-fructose group: Diet with eliminated sugar containing beverages, fruit juice and food items with high-fructose corn syrup (HFCS) as one of the top 5 ingredients on the label. Low-fat group: Diet based on the American Heart Association recommendations.	6mo	Oxidatized LDL-C	Changes in BMI z-score, BP, AST, ALT, HDL-C, LDL-C, TCHOL, TG, Urinary isoprostanes	NR
Ramon-Krauel et al, 2013 [27]	Child Obes.	USA	17	8 (Low-glycemic-load group)	9 (Low-fat diet group)	≥9% liver fat content assessed by MRS	Low-GL group: 11.8 ± 3.0. Low-fat group: 13.8 ± 3.2.	Low-glycemic load group: 7. Low-fat group: 7.	Low-GL group: Ad libitum diet. C:F:P: 40:35-40:20–25. Low to moderate GL carbohydrate food items. Low-fat group: Ad libitum diet. C:F:P:55-60:20:20–25. Vitamin supplements provided in both groups.	6mo	Intrahepatic tiglyceride content (IHTC)	Height, Body weight, waist/abdomen/hip circumferences, BP, TCHOL, HDL-C, LDL-C, TG, ALT, AST, Glucose, Insulin	94%
Jin et al, 2014 [28]	Nutrients	USA	24	13 (Glucose beverages group)	11 (Fructose beverages group)	IHTG content >8% assessed by MRS	Glucose beverages group: 14.2 ± 0.88. Fructose beverages group: 13.0 ± 0.71.	Glucose beverages group: 3. Fructose beverages group: 8.	Glucose beverages group: Consumption of 3 servings (8 fl oz bottles) of glucose-containing beverages daily. No diet or physical activity modification. Fructose beverages group: onsumption of 3 servings (8 fl oz bottles) of fructose-containing beverages daily. No diet or physical activity modification.	4wks	Body weight, Hepatic fat (%), ALT, AST, TG, FFAs, Glucose, Insulin, hs-CRP, LDL-C, lag time, oxLDL, PAI-1	NR	91.7%
Agustini et al, 2019 [29]	Med J Indones	Indo-nesia	32	16 (low-energy, GI, and fat)	16 (nutrition education)	US-diagnosed fatty liver	12-14yrs	24	Intervention group: Nutrition education and lunch diet (lunch box provided consisted of low-fat, <25% of total calories, low cholesterol, <300 mg/day, and low-GI diet). Control group: Nutrition education	12wks	FBG, Insulin, Body weight, Height, Dietary intake	NR	100%
Schwimmer et al, 2019 [30]	JAMA	USA	40	20 (Low-sugar diet group)	20 (Usual diet group)	≥10% of IHTG measured by MRI-PDFF and ALT≥45U/L	11-16yrs old. Low-sugar diet group: 12.8 ± 1.87. Usual diet group: 13.4 ± 1.9.	40	Low-sugar diet group: Avoidance of sugar-containing foods and drinks. Ad libitum diet with >3% of free sugars. Usual diet group: maintainance of their habitual diet.	8wks	Percentage of hepatic steatosis	Insulin resistance; ALT, AST, GGT, FBG, Insulin, TCHOL, LDL-C, HDL-C, TG, sweetness perception testing diet adherence, adverse events.	100%
Goss et al, 2020 [31]	Pediatr Obes.	USA	32	16 (CRD-group)	16 (FRD-group)	ALT>45IU/L and/or indication of echogenic liver via US	9–17 yrs old. CRD-group: 14.5 ± 2.6. FRD-group: 14.2 ± 2.1.	CRD-group: 9. RRD-group: 7.	CRD-group: Diet aiming at minimizing the intake of refined CHO. Meal plans provided. C:F:P:≤25:≥50:25. FRD-group: Diet promoting low-energy, high-quality foods, based on based on the USDA MyPlate Daily Food Plan. Meal plans provided. C:F:P:55:20:25.	8wks	Hepatic fat content, Body composition, TCHOL, LDL-C, HDL-C, TG, Glucose, Insulin, CRP, ALT, AST, GGT	NR	78%

Abbreviations: ALT—alanine aminotransferase; AST—aspartate aminotransferase; BMI—body mass index; BP—blood pressure; C:P:F—carbohydrates:protein:fats; CRD—carbohydrate-restricted diet; FBG—fasting blood glucose; FFAs—free-fatty acids; FRD—fat-restricted diet; GL—glycemic load; GGT—γ- glutamyl transferase; HDL-C—high-density lipoprotein cholesterol; hs-CRP—high sensitivity C-reactive protein; IHTG—intrahepatic triglycerides; LDL-C—low-density lipoprotein cholesterol; MRI-PDFF—magnetic resonance imaging (MRI) proton density fat fraction (PDFF); NAFLD—non-alcoholic fatty liver disease; oxLDL—oxidazide LDL; RCT—randomized clinical trial; TCHOL—total cholesterol; TG—triglycerides; and US—ultrasound.

**Table 2:** Characteristics based on lifestyle (i.e., diet plus exercise) and exercise-focused interventions.

Author [ref]	Journal	Country	Sample (n)	Intervention Group	Control Group	Nafld definition	Age (y) (mean ± SD)	Sex (boys)	Type of Intervention	Duration	Primary Outcomes	Secontary Outcomes	Completion Rates
**Diet plus exercise interventions**													
Wang et al, 2008 [32]	World J Gastroenterol	China	57	19 (Group 2)	38 (Group 1)	Liver fatty infiltration in US, abnormal ALT (1.5 times <50IU/L)	Group 1: 14.04 ± 1.8. Group 2: 13.4 ± 2.5.	Group 1: 26 Group 2: 13	Group 1: no intervention; Group 2: Aerobic exercise: 3 hrs of free exercise daily; Diet: Low-calorie (↓ 250 kcal)- high carbohydrate (50%)- low fat (10%) diet.	1mo	Height, Body weight, FBG, insulin, ALT, AST, TG, TCHOL, fatty liver indication in US	-	NR
Koot et al, 2015 [25]	International Journal of Obesity	The Netherlands	55	23 at Inpatient group & 21 at Ambulatory group	18 (Usual care group)	IHTG > 1.8% assesed by ^1^H-MRS	Inpatient treatment group: 14.9 ± 2.5. Ambulatory treatment group: 14.4 ± 2.1. Usual care group: 14.7 ± 2.4	Inpatient treatment group: 10. Ambulatory treatment group: 13. Usual care group: 10	Inpatient treatment group: High intensity aerobic exercise 4 days per wk (30–60min each); Nutrition (focused on dietary quality) and behavior (self-regulation, self-awareness, goal setting) modification sessions at weekly base (60min each). Ambulatory treatment group: Each session consisted of 60min high intensity exercise (plus advice to engage in exercise 3times/wk), an 1 hr-educational program and a 30min nutritional program Control group: Usual care	6mo & 18mo follow-up	^1^H-MRS determined liver steatosis, ALT	Length, Body weight, Waist circumference, BP, GGT, Insulin, HD-CL, LDL-C, Glucose	91% (6mo) and 80% (24mo)
Chan et al, 2018 [33]	Int J Obes (Lond)	China	52	26	26	IHTG≥5% measured by ^1^H-MRS	Intervention group: 15.3 ± 3.4. Control group: 13.8 ± 5.3.	Intervention group: 16. Control group: 17.	Intervention group: Individualized menu plan (balanced diet with a relative increase in protein) provided by a dietitian and exercise plan aiming at 30min aerobic exercise 2-3times/wk. Control group: Usual diet and exercise advice (↓ high-glycemic index carbohydrateand animal fat, and exercise for at least 2-3 times/wk for 30min per session.	16wks & 52wks follow-up	Changes in IHTC assessed by ^1^H-MRS at 16 wks	Maintanance of changes in IHTC at 52 wks, FBG, Lipid profile, ALT, AST, Serum insulin, Anthropometric measurements	80.8%
**Exercise-focused interventions**													
de Piano et al, 2012 [34]	Eur J Gastroenterol Hepatol	Brazil	28	14 aerobic training plus resistance training group (AT+RT)	14 aerobic training group (AT)	US-diagnosed fatty liver	16.48 ± 1.42	27	AT+RT group: Aerobic and resistance exercise training 3 times/wk for 1 h each time (30min aerobic training: running on treadmill, and 30min resistance trianing). AT-group: Aerobic exercise training 3 times/wk for 1 h each. Psychological intervention, Nutritional intervention (Nutritional education on a balanced diet), clinical evaluation once a week for both groups.	1yr	Body weight, Height, Body composition, FBG, Insulin, TCHOL, LDL-C, VLDL, HDL-C, TG, AST, ALT, GGT, Adipokines, Neuropeptides,	NR	NR

Abbreviations: ALT—alanine aminotransferase; AST—Aspartate aminotransferase; BMI—body mass index; GGT—γ- glutamyl transferase; HDL-C—high-density lipoprotein cholesterol; IHTG—intrahepatic triglycerides; LDL-C—low-density lipoprotein cholesterol; ^1^H-MRS—proton magnetic resonance spectroscopy; NAFLD—non-alcoholic fatty liver disease; RCT—randomized clinical trial; TCHOL—total cholesterol; TG—triglycerides; and US—ultrasound.

## Supplementary Materials

The following are available online at https://www.mdpi.com/2072-6643/12/9/2864/s1. Table S1: Quality of the included studies.
